# SP-IGAN: An Improved GAN Framework for Effective Utilization of Semantic Priors in Real-World Image Super-Resolution

**DOI:** 10.3390/e27040414

**Published:** 2025-04-11

**Authors:** Meng Wang, Zhengnan Li, Haipeng Liu, Zhaoyu Chen, Kewei Cai

**Affiliations:** 1 Faculty of Information Engineering and Automation, Kunming University of Science and Technology, Kunming 650500, China; wangmeng@kmust.edu.cn (M.W.); 20222204245@stu.kust.edu.cn (Z.L.);; 2 Yunnan Province Key Laboratory of Computer, Kunming University of Science and Technology, Kunming 650500, China

**Keywords:** single image super-resolution, semantic priors, long-range dependency, wavelet loss

## Abstract

Single-image super-resolution (SISR) based on GANs has achieved significant progress. However, these methods still face challenges when reconstructing locally consistent textures due to a lack of semantic understanding of image categories. This highlights the necessity of focusing on contextual information comprehension and the acquisition of high-frequency details in model design. To address this issue, we propose the Semantic Prior-Improved GAN (SP-IGAN) framework, which incorporates additional contextual semantic information into the Real-ESRGAN model. The framework consists of two branches. The main branch introduces a Graph Convolutional Channel Attention (GCCA) module to transform channel dependencies into adjacency relationships between feature vertices, thereby enhancing pixel associations. The auxiliary branch strengthens the correlation between semantic category information and regional textures in the Residual-in-Residual Dense Block (RRDB) module. The auxiliary branch employs a pretrained segmentation model to accurately extract regional semantic information from the input low-resolution image. This information is injected into the RRDB module through Spatial Feature Transform (SFT) layers, generating more accurate and semantically consistent texture details. Additionally, a wavelet loss is incorporated into the loss function to capture high-frequency details that are often overlooked. The experimental results demonstrate that the proposed SP-IGAN outperforms state-of-the-art (SOTA) super-resolution models across multiple public datasets. For the X4 super-resolution task, SP-IGAN achieves a 0.55 dB improvement in Peak Signal-to-Noise Ratio (PSNR) and a 0.0363 increase in Structural Similarity Index (SSIM) compared to the baseline model Real-ESRGAN.

## 1. Introduction

The goal of SISR is to recover high-frequency information that is missing from low-resolution images, a task that poses significant challenges. SISR has a wide range of practical applications requiring high-resolution images, such as surveillance image enhancement [1], satellite remote sensing data processing [2], and identity recognition. Traditional super-resolution methods [3] are often constrained by algorithmic resolution limits and unrealistic detail recovery, making them inadequate for practical applications.

In recent years, SISR methods have been developed based on convolutional neural networks (CNNs) [4] and generative adversarial networks (GANs) [5], among others. For example, SRCNN [6] was the first to introduce deep convolutional neural networks to learn the mapping from low-resolution to high-resolution images, incorporating pixel loss (L1 loss and Mean Squared Error (MSE) loss). Models such as VDSR [7], EDSR [8], and RCAN [9] increased network depth and introduced mechanisms like residual learning to mitigate the issue of gradient explosion while enhancing the network’s depth. Subsequently, various CNN design frameworks [6,10] have been proposed to enhance the representational capacity of super-resolution networks through pixel-level loss constraints. Although these methods improve reconstruction quality, the generated textures are overly smooth and lack sufficient high-frequency details. To better meet subjective visual quality requirements [11], more detailed and visually appealing textures are needed. Inspired by generative adversarial networks (GANs) [12], Christian Ledig et al. proposed SRGAN [13], which was the first model to incorporate perceptual loss into the original loss function, placing greater emphasis on visual texture details. ESRGAN [14], proposed by Wang et al, is an improved version of SRGAN. It incorporates Residual-in-Residual Dense Blocks (RRDBs) and a Relativistic Discriminator, which not only enhance image quality but also preserve important details. A series of GAN-based models [15,16,17] have advanced SR networks in their generation of fine-grained semantic textures. However, GAN-based models often produce unrealistic textures and unwanted artifacts during adversarial training when generating high-resolution images.

With the rapid advancement of deep learning, the application scope of single-image super-resolution (SISR) has gradually expanded to real-world scenarios. However, traditional degradation processes are often simplified into known standard models, such as those in [6,13], to generate low-resolution input images. Nonetheless, super-resolution methods based on fixed degradation models [3,4,6,12] face significant challenges when dealing with the more complex degradation processes of real-world images. To address this issue, Fritsche et al. proposed a real-world degradation model [18], which improves the traditional degradation process in SRGAN by incorporating real-world factors such as noise, blur, and JPEG compression, significantly enhancing the model’s generalization ability. Additionally, the Real-ESRGAN model proposed by Wang et al. [19] introduced a high-order degradation model that simulates more realistic image degradation processes. By incorporating real-world image data during training, it further improves the model’s generalization ability, enabling it to learn and reconstruct richer, more detailed image textures. The DASR model [20] employs an adaptive degradation mechanism, excelling at handling unpredictable degradation in real-world images and effectively recovering more realistic image details. However, these methods still face two major issues: (1) in complex scenarios, models are more prone to inconsistent local texture reconstruction due to a lack of semantic understanding; (2) it has yet to be determined how we can better leverage contextual information to improve texture generation and reduce artifacts.

In summary, this paper extends the GCCA module to the Real-ESRGAN model with semantic information. The introduction of semantic priors enhances the model’s understanding of image structure, while abstracting convolutionally extracted texture information into feature representations. The texture data from previous layers are treated as feature vertices, with their relationships represented by an adjacency matrix, enabling contextual dependencies. This allows the model to capture long-range contextual relationships, effectively utilizing semantic information to reduce semantic inconsistencies in reconstructed images. Additionally, incorporating wavelet loss into the total loss function further enhances the model’s ability to capture high-frequency details and reduce artifacts. The proposed branch structure, SP-IGAN, enables the model to fully understand the generated image structure and type, preventing information confusion and producing realistic detailed information. The contributions of this paper are summarized as follows:

- The Spatial Feature Transform (SFT) layer is introduced into the Residual-in-Residual Dense Block (RRDB) module. The pre-trained segmentation network in the auxiliary branch accurately extracts regional semantic information, which is then conditioned and input into the RRDB module through the SFT layer via affine transformations. This provides the model with additional contextual semantic understanding, enhancing the correlation between texture information and category information, which leads to more accurate and semantically consistent texture reconstruction. Additionally, a Star-shaped Residual-in-Residual Dense Block (StarRRDB) [21] is employed, which offers higher network capacity compared to the RRDB module used in Real-ESRGAN. The design of StarRRDB is inspired by ESRGAN+ [22], providing greater flexibility and potential for the integration of the SFT layer and the subsequent GCCA module.
- The Graph Convolutional Channel Attention (GCCA) module enhances the RRDB module by transforming channel dependencies into adjacency relationships of feature vertices, thereby improving pixel correlation. The GCCA module draws on the theory of graph neural networks, focusing on the attention mechanism for feature interaction between channels. It effectively highlights key features, suppresses secondary features, reduces computational redundancy, and enhances the model’s ability to capture long-range dependencies.
- Add the wavelet loss [23] to the overall loss function. Wavelet loss effectively captures high-frequency details that are often overlooked by conventional pixel-based loss functions. By combining wavelet loss with the standard GAN loss, the model can more accurately recover high-frequency components, thereby generating images with richer textures and finer details.

## 2. Related Work

### 2.1. Single-Image Super-Resolution Method Incorporating the Attention Mechanism

The attention mechanism [24] simulates human cognitive processes, effectively focusing on key information and re-weighting the features in the network. However, when processing outdoor scenes and real-world images with complex textures, the generated textures often lack realism, and the utilization of long-range contextual information is insufficient. Therefore, it is crucial to guide the model in understanding the relevance of long-range texture information through the attention mechanism. In recent years, many attention-based networks have been proposed to address various visual tasks and have been widely applied in CNNs. For example, Hu et al. first introduced SENet [25], which introduced a more modular attention mechanism compared to other networks. Zhang et al. introduced the attention mechanism into the image super-resolution task, incorporating the Channel Attention (CA) mechanism in their proposed RCAN [9] to focus attention on more important channels. Buades A et al. employed the Non-Local (NL) [26] module, calculating the response at a specific location as the weighted sum of features from all locations. Woo and Park used the CBAM [27] to enhance the expressive power of channel and spatial features. Furthermore, Dai et al. proposed the Second-order Attention Network (SAN) [28], which incorporates long-range dependencies and global information from the entire network structure. This method enables the model to effectively leverage information across different scales and levels when capturing image details, resulting in significant performance improvements.

### 2.2. Single-Image Super-Resolution Method Incorporating Prior Information

Traditional super-resolution methods typically rely on a single low-resolution image for reconstruction. However, introducing auxiliary conditional information provides the generative network with a richer understanding of the contextual information, enabling more detailed image reconstruction. Wang et al. first introduced the SFT layer in the SFTGAN [29], integrating semantic probability maps into the network. This allows the model to generate corresponding texture features during image generation, thereby enhancing the realism of textures. The SROOE [30] proposed by Park et al. predicts the optimal target image as conditional information to be input into the generative model, which, through training on the target trajectory, generates more credible results. The FxSR [31] incorporates multi-task learning, training the model with combinations of various loss functions as conditional information. By introducing different weightings for perceptual loss, GAN loss, and L1 loss for different features, it significantly reduces artifact generation. Ma et al. proposed the SPSR model [32], which utilizes gradient information of images to guide the super-resolution process. The model employs a gradient branch to restore high-resolution gradient maps, thereby providing structural priors for the super-resolution process. Additionally, a gradient loss is introduced to impose a second-order constraint, which reduces geometric distortions while preserving image structure and details, resulting in more natural and realistic generated images. Li et al. proposed SeD [33], which incorporates semantic information of the image as a condition into the discriminator, encouraging the super-resolution network to learn a more refined distribution. These studies clearly demonstrate that incorporating additional conditional information significantly enhances the model’s understanding of image content. Overall, the introduction of conditional information provides new insights and methods for super-resolution techniques, driving further improvements in image reconstruction quality.

## 3. Methods

### 3.1. Network Architecture

**Figure 1:**
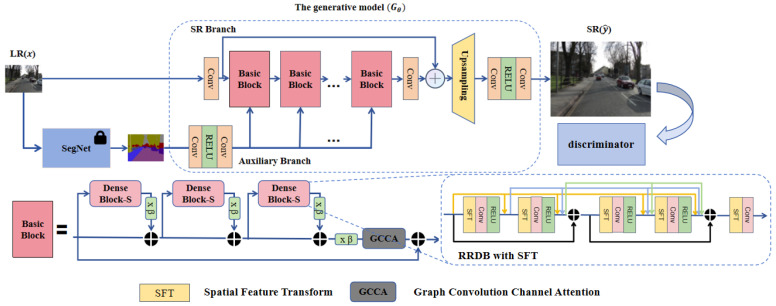
The overall structure of SP-IGAN consists of three core modules: Spatial Feature Transform (SFT) Layer, Graph Convolutional Channel Attention (GCCA), and Residual-in-Residual Dense Block (RRDB). Additionally, a star-shaped residual connection is used to provide greater flexibility for future extensions.

To address the issue of inconsistent local texture reconstruction in images due to the lack of categorical semantic understanding, we propose a GAN model branch structure, SP-IGAN, that effectively utilizes semantic priors. Using Real-ESRGAN as the baseline model, we introduce additional semantic information as auxiliary input. The overall model structure is shown in Figure 1, and its core components include the Spatial Feature Transform (SFT) Layer, Graph Convolutional Channel Attention (GCCA), and Residual-in-Residual Dense Block (RRDB). The entire framework consists of two branches: the main branch and the auxiliary branch.

The main branch introduces the GCCA module, which converts channel dependencies into adjacency relationships between feature vertices, thus enhancing pixel correlations. Additionally, the auxiliary branch strengthens the dependence between class semantic information and regional texture within the RRDB module. Furthermore, we employ StarRRDB [21] to expand the network capacity of RRDB.

The auxiliary branch employs the pre-trained segmentation model OutdoorSceneSeg, which is used in the SFTGAN [29] model, to generate semantic maps. This model accurately extracts region-specific semantic information from the input low-resolution images and injects it into the RRDB module through the SFT layer. During this process, the Non-Local (NL) operation [26] is used to treat the feature maps of previous and subsequent layers as an adjacency matrix, establishing contextual dependencies. This allows the model to capture long-range contextual information, while the SFT layer injects additional semantic information, enhancing its utilization efficiency. The following sections will detail the specific design and implementation of the related modules.

### 3.2. The RRDB Structure with the SFT Layer

**Figure 2:**
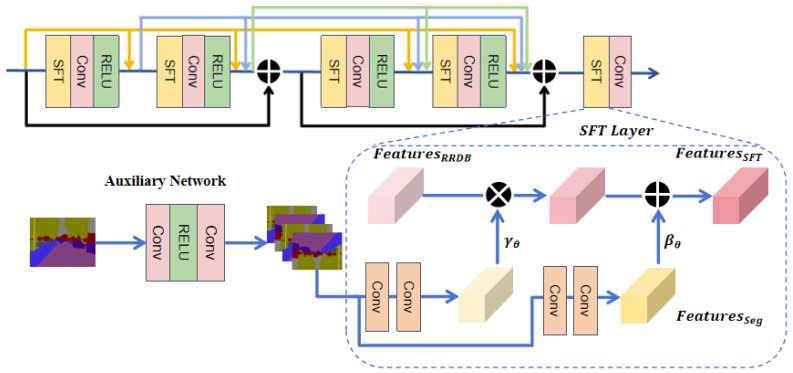
The SFT layer transforms semantic information through a mapping function. The auxiliary network generates auxiliary semantic information and shares it with all SFT layers to produce modulation parameters.

The RRDB module adopts a residual structure, using dense residual connections between layers to enhance feature reuse efficiency and model stability, while simultaneously reducing the number of parameters.

(1) $$F_{RRDB}=x+F\left(x,\theta \right)$$

where F(x,θ) represents the nonlinear transformation of low-resolution image $x\in R^{C\times H\times W}$ by the RRDB module, where θ denotes network parameters. Inspired by [30,31], the introduction of additional conditional information significantly enhances the model’s understanding of image content. We integrate the SFT layer into the RRDB module, where the SFT layer performs spatial transformations on features by introducing a pretrained semantic map, thereby improving the network’s ability to comprehend image content. The specific network architecture is shown in Figure 2. The SFT layer learns a mapping function $f_{M}\left(\cdot \right)$, which includes a set of parameters (γ,β) and semantic prior information Φ. Spatial affine transformations are applied to the intermediate features at each layer.

(2) $$y=G_{\theta }\left(x|\gamma ,\beta \right),\;\left(\gamma ,\beta \right)=f_{M}\left(\Phi \right)$$

Here, the prior information Φ is first used to construct a pair of affine transformation parameters M:Φ→(γ,β) through the mapping function (γ,β), where (γ,β) represents the scaling and translation factors, and the input variable $x\in R^{C\times H\times W}$ denotes the low-resolution image. The mapping function $f_{M}\left(\cdot \right)$ serves to convert the prior information Φ into affine transformation parameters (γ,β), which are then used for further affine transformation processing of the input image.

(3) $$F_{SFT}\left(F_{RRDB}|\gamma ,\beta \right)=\gamma ⊙F_{RRDB}+\beta$$

where ⊙ represents element-wise multiplication, where $F_{RRDB}$ is the intermediate feature to be modulated, $\gamma \in R^{C\times H\times W}$ and $\beta \in R^{C\times H\times W}$ are the two modulation parameters predicted by the auxiliary network. In the main branch, the dimensions of the feature map are aligned with parameters γ and β, and fusion is performed via element-wise multiplication. The global semantic features are then extracted through the feature extraction module ε.

(4) $$F_{Global}=\varepsilon \left(x;\gamma ,\beta \right)$$

where ε(·) is a function that fuses the input image features with the modulation parameters, achieving both spatial transformation and feature fusion. Additionally, multiple SFT layers share parameters, which enhances the efficiency of semantic information transfer. This design allows for fine-grained control of features, enabling the network to better capture the structural and texture information of the image.

### 3.3. The RRDB Module Integrated with GCCA Attention

**Figure 3:**
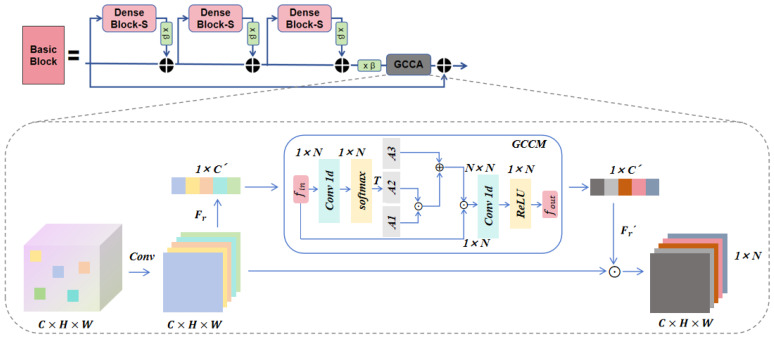
The framework of GCCA extended to the RRDB module consists of two parts. The first part involves the mapping operations represented by $F_{r}$ and ${F_{r}}^{\prime }$, where ⊙ denotes element-wise multiplication. The second part is the GCCM (Graph Convolutional Channel Module), where *T* represents the diagonal arrangement result.

Although star-shaped dense residual connections can more effectively learn features and stabilize the training of deep networks, the interdependencies between layers have not been fully exploited. After introducing semantic information, the network needs to better understand the relationships between image features, improving its representational and perceptual abilities. Inspired by the design in [34], we propose extending GCCA attention to the RRDB module, as shown in Figure 3. The GCCA module performs non-local (NL) operations [26] directly on the features, enabling better capture of long-range dependencies in the image, improving the understanding of global context, and assigning different weights to the feature map.

#### 3.3.1. NL Operations

First, the definition of non-local (NL) [26] operations in CNNs is as follows:

(5) $$f_{out}\left(a,b\right)=\sum_{(\tilde{a},\tilde{b})\in L}\frac{1}{N(\tilde{a},\tilde{b})}w\left(\tilde{a},\tilde{b}\right)f_{in}\left(\tilde{a},\tilde{b}\right)$$

where $f_{in}\left(\cdot \right)$ and $f_{out}\left(\cdot \right)$ represent the input and output feature maps, respectively, while (a,b) and $\left(\tilde{a},\tilde{b}\right)$ denote the horizontal and vertical coordinates of the pixels on the feature map. $w(\tilde{a},\tilde{b})$ is the weight corresponding to the convolutional layer, representing the relationship between input and output. *L* is the 3×3 convolutional region for the NL operation, which includes (a,b) and eight adjacent pixels. Therefore, the output at each position aggregates all information related to (a,b) and its neighboring pixels. N(·) denotes the normalization operation applied to $\left(\tilde{a},\tilde{b}\right)$.

Considering the intentional representation of spatial encoding in NL operations, the channel attention module utilizes the channel weights computed based on spatial information to adjust the response intensity of each channel. However, focusing on each corresponding pixel significantly increases computational complexity and leads to redundant calculations, which negatively affects the reconstruction results. To address this, we use Graph Convolutional Networks (GCNs) [35] to directly perform NL operations on the features. Therefore, the GCNs operation on the feature vertex $i_{\theta }$ can be expressed as follows:

(6) $$f_{out}\left(i_{a}\right)=\sum_{i_{b}\in C_{a}}\frac{1}{N(i_{b})}W_{ab}f_{in}\left(i_{b}\right)$$

where *a* and *b* represent the indices of the vertices in the feature map, while *i* denotes a vertex in the feature map. $C_{a}$ represents the convolutional sampling set of the output vertex $i_{a}$ and $i_{b}$ refers to the set of vertices adjacent to vertex $i_{a}$. $w_{ab}$ is the corresponding weight vector, which represents the relationship between each output vertex $i_{a}$ and input vertex $i_{b}$. N(·) is used to balance and normalize each subset.

This allows the model to focus more on the corresponding features rather than the pixels, effectively reducing feature redundancy. We employ a Graph Convolutional Module (GCCM) to capture the adjacency relationships of different features across layers, enabling NL operations on the features. This allows the construction of interdependencies between layers, enhancing the representational power of the features.

#### 3.3.2. Graph Convolution Channel Attention (GCCA)

We integrate the GCCA module into each Basic Block, performing NL operations on the features directly after semantic information is extracted through the SFT layer in the SP-IGAN model. The GCCA module consists of two parts, as shown in Figure 3. The first part is a feature mapping layer formed by two linear embedding functions f(·), while the second part is the Graph Convolutional Module (GCCM), which extracts feature weights *W*. The design is inspired by the bottleneck structure [36,37]. On one hand, this structure effectively enables channel interaction, generating interdependencies. On the other hand, it has been shown to reduce feature redundancy, thereby enhancing model performance. Consequently, the bottleneck structure replaces the convolutional layers, reducing the number of convolutional layers from C×C to $C\times (\frac{C}{\omega })$, compressing the adjacency matrix size to $\left(\frac{C}{\omega }\right)\times \left(\frac{C}{\omega }\right)$, with a compression factor of ω, to reduce the parameter count.

The workflow of the GCCA module can be divided into several steps: First, global features from each channel of the input feature map $f_{in}$ are extracted using Global Average Pooling (GAP) [38], compressing spatial dimension $F_{C\times H\times W}$ into the channel dimension $F_{C\times 1\times 1}$, where *C* represents the number of channels, which is also the number of feature vertices *i* in the GCCA module. Next, the global features are used to generate channel weights *W*. The feature mapping layer consists of a linear embedding function $f_{out}=Wf_{in}$, where *W* is a learnable weight matrix. The mapping is performed using 1×1 convolution. The weight of each vertex is generated by the Graph Convolutional Module (GCCM), and these weights are then mapped back to the initial feature map $f_{in}$, as the weights for the corresponding channel *C*. Finally, the weight for each channel is calculated using the Sigmoid activation function. These channel weights, *W*, are element-wise multiplied with the original feature map $f_{in}$, to adjust each channel.

The introduction of the GCCA module enables the model to more effectively capture key features while suppressing irrelevant ones, thereby improving overall performance. The correlation and importance between channels have a significant impact on the final outcome. By adaptively adjusting the channel weights, the GCCA module significantly enhances the model’s expressive power and generalization ability. The formula for the GCCA module is as follows:

(7) $$\left\{\begin{array}{cc} y^{\prime } & =x^{\prime }\cdot sigmoid\left({F_{r}}^{\prime }\left(ReLU(G\left(x^{\prime \prime },M\right))\right)\right)\\ x^{\prime \prime } & =F_{r}\left(GAP\left(x^{\prime }\right)\right) \end{array}\right.$$

The two embedding functions, ${F_{r}}^{\prime }\left(\cdot \right)$ and $F_{r}\left(\cdot \right)$, form a bottleneck structure, where $F_{r}\left(GAP\left(x^{\prime }\right)\right)$ serves as the input to the GCCM, and *M* represents an adjacency matrix that captures the relationships between features. $x^{\prime }$ and $y^{\prime }$ denote the input and output feature maps of the GCCA module, respectively. Since each feature node in a GCN should be independent [39], the feature nodes in the GCCA module are particularly sensitive to feature redundancy. When two feature nodes are similar or identical, their relationships are often difficult to accurately express in the adjacency matrix. Therefore, the bottleneck structure plays a crucial role in the GCCA module. Meanwhile, the GCCM module can be viewed as guiding attention through the GCNs, enabling the module to adaptively learn the interdependencies between feature vertices, thereby more effectively capturing the correlations between nodes.

#### 3.3.3. Graph Convolutional Channel Module (GCCM)

In GCNs, the feature map is mapped to F×N, where *F* represents the number of features per vertex and *N* denotes the number of vertices. Therefore, Equation (7) is rewritten as follows:

(8) $$f_{out}=Wf_{in}\tilde{A}$$

The matrix $\tilde{A}$ of N×N represents the normalization operation on the adjacency matrix, which can be defined as follows: $\tilde{A}=D^{-\frac{1}{2}}AD^{-\frac{1}{2}}$, $\tilde{A}$ is the adjacency matrix of N×N, and *D* denotes the degree matrix, defined as $D_{aa}=\sum_{b}A_{ab}+\epsilon$, where ϵ is the small value used to prevent division by zero. $A_{ab}$ represents the connection between vertex *a* and vertex *b*. Due to the varying feature distributions across layers, the relationships between features are difficult to capture directly. Therefore, we adopt this adaptive, learnable graph convolution structure, which automatically learns the dependencies between feature vertices a∼b based on the data. Therefore, Equation (8) is rewritten as follows:

(9) $$f_{out}\left(x\right)=Wf_{in}\left(A_{1}\times A_{2}+A_{3}\right)$$

The adjacency matrix is composed of three parts: $A_{Adj}=\left\{A_{1},A_{2},A_{3}\right\}$; $A_{1}$ represents the identity matrix of N×N, capturing the information of the feature vertex itself. From Equation (10), it follows that ${\tilde{A}}_{1}=D^{-\frac{1}{2}}A_{1}D^{-\frac{1}{2}}=A_{1}$ remains the identity matrix;

$A_{2}$ represents a diagonal matrix of N×N, where the weights for each feature vertex a∼b are calculated through the self-attention mechanism. The self-attention map enables the network to adaptively emphasize or suppress features. The calculation process of the self-attention map is as follows:

(10) $$A_{2}=T\left(softmax\left(Wf_{\mathrm{in}}\right)\right)$$

where *T* represents arranging the results into a diagonal matrix, and *W* denotes the weights of the one-dimensional convolutional layer;

$A_{3}$ represents the adjacency matrix of N×N, generating the relationships between any two feature vertices during training, and customizing the dependencies for various feature vertices contained in different layers.

The adjacency matrix $A_{Adj}=\left\{A_{1},A_{2},A_{3}\right\}$ is used to describe the relationships between different feature groups in the convolutional neural network structure. These relationships are progressively constructed through adaptive learning. Subsequently, the adjacency matrix $A_{Adj}$ is used to update the feature vertices, and the updated feature vertices serve as channel weights, enabling precise calibration of channel-level responses. In the channel attention mechanism, this design precisely adjusts the importance of features, effectively capturing key features and significantly enhancing the model’s expressive power and performance.

### 3.4. Loss Functions

**Figure 4:**
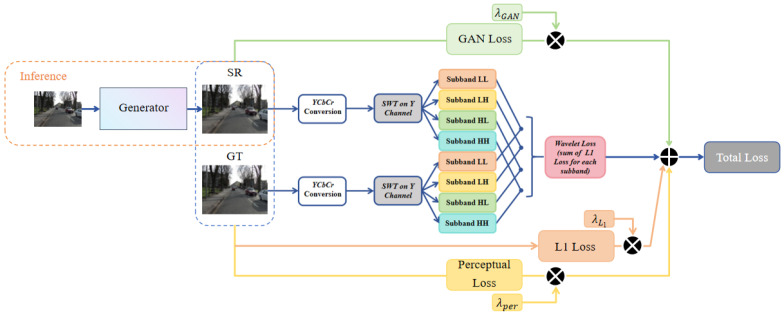
The structure diagram of the discriminator, which includes wavelet loss $L_{SWT}$.

Most GAN-based super-resolution models typically adopt a strategy that combines L1 loss [6], perceptual loss [13], and GAN loss [12]. L1 loss improves the overall image fidelity by reducing the mean absolute error between the predicted and true image pixel values, as given by the following formula: $L_{L1}=\frac{1}{N}\sum_{i=1}^{N}∥{\hat{y}}_{i}-y_{i}∥$. Perceptual loss strengthens feature representations related to human perception by aligning features extracted from a pre-trained network, as given by the following formula: $L_{perceptual}=\frac{1}{M}\sum_{i=1}^{M}{∥\varphi \left({\hat{y}}_{i}\right)-\varphi \left(y_{i}\right)∥}_{2}^{2}$. GAN loss focuses on generating results that approximate the real image, significantly enhancing the realism of image textures. The formulas are as follows: Generator loss: $L_{G}=-E_{x∼p_{z}}\left[\log D(G(z))\right]$, Discriminator loss: $L_{D}=-E_{x∼p_{data}}\left[\log D(x)\right]-E_{x∼p_{z}}\left[\log(1-D(G(z)))\right]$. In real-world super-resolution, capturing more visually realistic images requires focusing on high-frequency details. Inspired by [40], we introduced wavelet loss into the loss function of the Real-ESRGAN model. Wavelet loss is evaluated by calculating the L1 fidelity difference between the generated and true images in the SWT subbands. The principle of wavelet loss is illustrated in Figure 4.

The specific steps for wavelet loss are as follows: Apply wavelet transform SWT(·) to the generated image *G* and the true image *X*, converting from the RGB space to the YCbCr space. Then, perform wavelet transform SWT(·) to decompose the Y channel into different frequency subbands, as expressed below:

(11) $$\left\{\begin{array}{cc} SWT(G) & =\{G_{L},G_{H}\}\\ SWT(X) & =\{X_{L},X_{H}\} \end{array}\right.$$

Here, SWT(·) decomposes the Y channel of the image into one low-frequency (LF) subband, denoted as $G_{L}=\left\{G_{LL}\right\},X_{L}=\left\{X_{LL}\right\}$, and multiple high-frequency (HF) subbands, named $G_{H}=\left\{G_{LH},G_{HL},G_{HH}\right\},X_{H}=\left\{X_{LH},X_{HL},X_{HH}\right\}$, collectively forming the output. The specific implementation of the wavelet loss function is as follows:

(12) $$L_{SWT}=E\left[\sum_{j}\lambda_{j}{∥SWT{\left(G\left(x\right)\right)}_{j}-SWT{\left(X\right)}_{j}∥}_{1}\right]$$

where G(·) represents the generator model of the proposed SP-IGAN structure, and $\lambda_{j}$ is the scaling factor that controls the high-frequency details generated. Therefore, our total loss function incorporates wavelet loss in addition to the original $L_{1}$ loss, perceptual loss, and GAN loss, and can be expressed as follows:

(13) $$L=\lambda_{1}\cdot L_{1}+\lambda_{per}\cdot L_{per}+\lambda_{GAN}\cdot L_{GAN}+\lambda_{SWT}\cdot L_{SWT}$$

Traditional pixel-level loss functions are ineffective at capturing high-frequency details in images. By incorporating wavelet loss, the model’s ability to reconstruct high-frequency details is enhanced, addressing this limitation.

## 4. Experiments

This chapter first introduces the experimental setup, including the datasets and test sets used, implementation details, and the computation methods for evaluation metrics. It then analyzes the impact of the proposed SP-IGAN structure on the final reconstruction results under different classification priors. Subsequently, the SP-IGAN structure is compared with SOTA methods on the dataset, and finally, an ablation study is conducted on the model.

### 4.1. Experimental Setup

#### 4.1.1. Datasets

To begin with, it is important to define high-resolution (HR) and low-resolution (LR) images. HR and LR are relative concepts, and the specific resolution values depend on the application scenario. High-resolution images typically refer to the original, unprocessed images, characterized by rich details, sharp edges, and a high pixel density. These images are usually the ones in the dataset and serve as the ground truth in training data, paired with low-resolution images. In contrast, low-resolution images are derived from high-resolution images through a degradation model, which may include downsampling, noise, blurring, or JPEG compression. The purpose of this process is to reduce the image details and resolution, simulating real-world degradation scenarios, and these low-resolution images are used as input for super-resolution models. They generally have fewer pixels and exhibit blurred details. Super-resolution tasks are often defined by a scaling factor (e.g., ×2, ×4, ×8), indicating the target magnification, and thus there is a certain multiplicative relationship between HR and LR images. We trained the model using the DF2K and OST datasets, where DF2K is a combined dataset consisting of DIV2K and Flickr2K. DIV2K contains 800 images, Flickr2K provides 2650 images, and the OST (Outdoor Scene Train/Test) dataset includes 300 high-resolution outdoor scene images. The pre-trained segmentation model used is OutdoorSceneSeg, which is employed in the SFTGAN [29] model. It is pre-trained on the COCO dataset and further fine-tuned on the ADE20K dataset. This segmentation model is capable of distinguishing 8 categories (7 object categories, including building, grass, plant, water, mountain, sky, and animal, along with 1 background category). For undefined categories, the model classifies them as “background”. The number of category labels can be flexibly adjusted according to the specific application scenario and task requirements. The test set includes three public benchmark datasets for single-image super-resolution tasks: DF2K, Set5, BSD100, and the outdoor dataset OST.

#### 4.1.2. Implementation Details

The training process is similar to Real-ESRGAN, with high-resolution images set to 256 × 256 and a total batch size of 48. The SP-IGANNet model is first trained using L1 loss, with the L1 loss derived from the pre-trained ESRGAN model, for 100K iterations. The SP-IGANNet model is then initialized as the generator, and SP-IGAN is trained for 50K iterations using a combination of L1 loss, perceptual loss, GAN loss, and wavelet loss. We applied exponential moving average (EMA) to stabilize the training process and achieve better performance. Additionally, we visually enhanced the sharpness of the images using the Unsharp Masking (USM) method. During training, the real images are sharpened, which helps enhance image clarity while effectively suppressing artifacts caused by over-enhancement. The model is trained using the sharpened real images. The entire process was conducted on an RTX 3060 GPU with a batch size of 2, a convolution kernel size of 3 × 3, and a stride of 1. The Adaptive Moment Estimation (Adam) optimizer was used, with an initial learning rate of 0.0002 and an exponential moving average decay factor of 0.999. The model was trained under the PyTorch (https://pytorch.org/) framework.

#### 4.1.3. Evaluation Index

In image super-resolution, Peak Signal-to-Noise Ratio (PSNR) is commonly used as a metric to evaluate the quality of image reconstruction. A higher PSNR value indicates better image quality. PSNR is calculated based on the Mean Squared Error (MSE) of the image, as shown in the following formula:

(14) $$PSNR=10\cdot \log_{10}\left(\frac{R^{2}}{MSE}\right)$$

where MSE represents the difference between the predicted and ground truth values of an image, and is calculated using the following formula:

(15) $$MSE=\frac{1}{N}\sum_{i=1}^{N}{\left({\hat{y}}_{i}-y_{i}\right)}^{2}$$

The Structural Similarity Index (SSIM) is a structure-based image quality metric designed to simulate structural similarity in human visual perception. SSIM measures visual information, including pixel differences, luminance, contrast, and structure, and better aligns with human visual perception compared to PSNR. The formula is as follows:

(16) $$SSIM\left(x,y\right)=\frac{\left(2\mu_{x}\mu_{y}+C_{1}\right)\left(2\sigma_{xy}+C_{2}\right)}{\left(\mu_{x}^{2}+\mu_{y}^{2}+C_{1}\right)\left(\sigma_{x}^{2}+\sigma_{y}^{2}+C_{2}\right)}$$

The value of SSIM ranges from 0 to 1, where a value closer to 1 indicates greater similarity between two images and higher image quality.

### 4.2. The Effect of Low-Resolution Images on Segmentation Models

**Figure 5:**
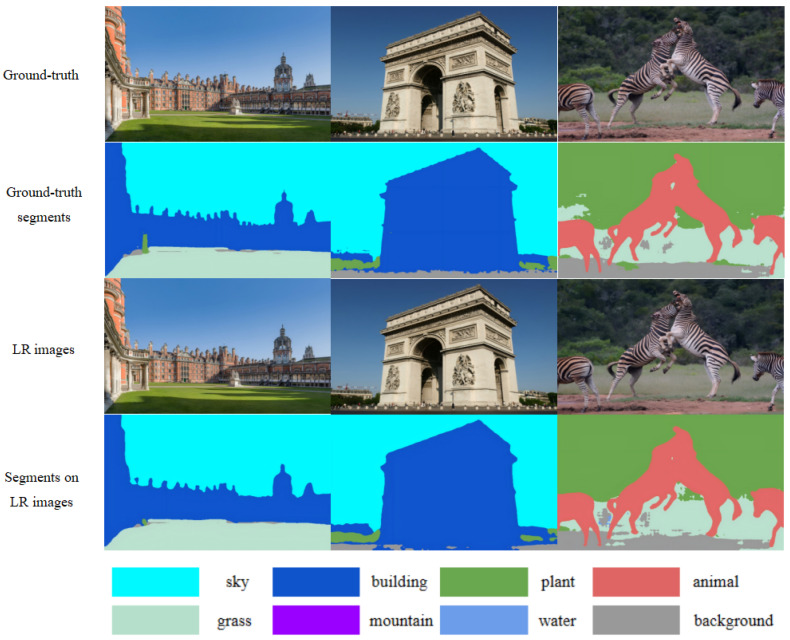
Examples of segmentation are shown as follows: Row 1: Ground Truth (GT) image; Row 2: Segmentation result of GT; Row 3: low-resolution (LR) image; Row 4: segmentation result of LR.

In super-resolution research, low-resolution (LR) images are obtained by downsampling high-resolution (HR) images. Therefore, in our experiments, we need to consider whether there is a significant difference between the semantic maps derived from HR and LR images through the segmentation model, or if low-resolution images are too affected to provide accurate results, as shown in Figure 5. We found that even at this low resolution, the semantic maps obtained through segmentation are not significantly affected. The segmentation results from LR and HR images are very similar, but this is mainly true for larger and more distinct objects; smaller objects were not tested.

### 4.3. Effect of Different Priors on the Reconstruction Results

**Figure 6:**
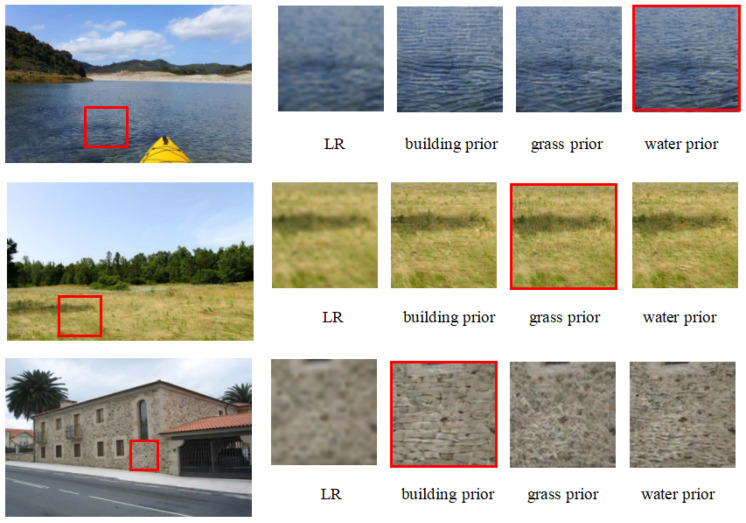
Reconstruction results under priors from different categories.

In the experiment, we need to analyze whether prior information influences the output reconstruction results. As shown in Figure 6, under the influence of different categories of prior information, a given LR image can be reconstructed to match the texture corresponding to the guided prior. We can observe that for the water category in the lake, when guided by prior information from buildings and grass, the reconstructed texture matches that of the corresponding category. Similarly, guidance from prior information of grass and buildings also leads to the corresponding textures being generated. Regardless of the input category, the prior for the building category always results in the generation of a regular geometric texture. This phenomenon demonstrates the importance of semantic priors in reconstructing real textures in single-image super-resolution tasks. In the absence of semantic priors, the model may produce reconstructions that are inconsistent with the real texture.

### 4.4. Comparison with State-of-the-Art Methods

#### 4.4.1. Quantitative Results

We compared the SSIM and PSNR metrics to evaluate the model’s performance, as shown in Table 1. Although the PSNR metric measures image reconstruction quality to some extent, it cannot fully reflect human perceptual preferences. Therefore, we also computed the SSIM metric, which better reflects the subjective quality of human visual perception. Compared to recent GAN-based super-resolution models, our model demonstrates significant superiority. For example, on the ×4 super-resolution of the DF2K test set, our model achieved a 0.55 dB improvement in PSNR over the baseline Real-ESRGAN model, and a 0.0363 improvement in SSIM, validating the effectiveness of our proposed solution. Although Real-ESRGAN [19] and IG-CFAT [40] excel in restoring high-frequency details, they tend to generate unnatural textures, making it difficult to ensure the accuracy of the generated textures. In contrast, our approach introduces a semantic prior conditional network, providing features for each category to the generation network, making the generated textures more natural and realistic. Compared to the SFTGAN [29] model, which also uses semantic priors, our model shows a 2.22 dB improvement in PSNR and a significant advantage in SSIM, with an increase of 0.1308.

#### 4.4.2. Qualitative Results

**Figure 7:**
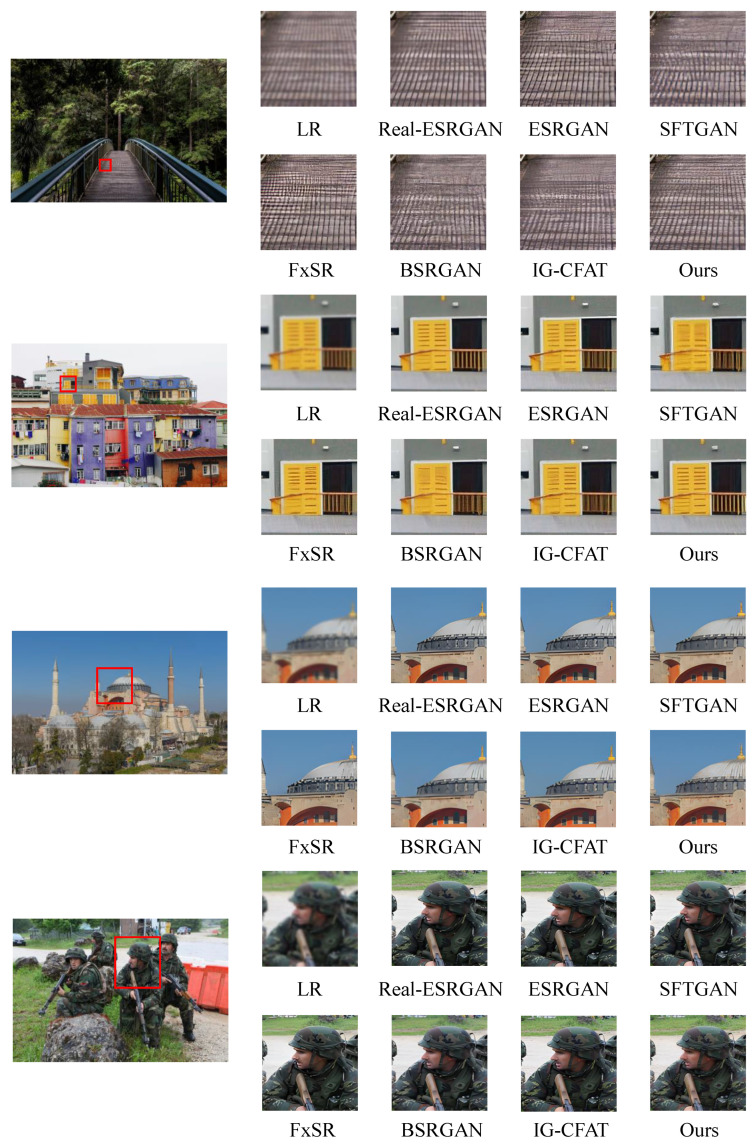
Visual comparison of SP-IGAN ×4 and other super-resolution methods.

To demonstrate the performance of our model, Figure 7 presents a comparative analysis with several state-of-the-art (SOTA) models in real-world image super-resolution, including Real-ESRGAN [19], SFTGAN [29], BSRGAN [17], IG-CFAT [40], and ESRGAN [14]. A comprehensive comparison was conducted on the performance of these models under ×2, ×4, and ×8 super-resolution scenarios. All experiments were conducted on the same dataset, DIV2K, and the qualitative results are presented in Figure 7. As observed, compared to other models, our model reconstructs details more naturally and realistically, preserving texture characteristics closer to those of real images. The first three images depict scenes with architectural texture details, where the model accurately restores design details and corresponding textures, producing structures highly similar to the original images. In the last image, the model also clearly reconstructs the soldier’s features, further demonstrating its superiority in fine-detail reconstruction.

### 4.5. Ablation Study

To better assess the effectiveness of SP-IGAN, we conducted ablation experiments on the StarRRDB connection, GCCA module, SFT layer, and wavelet loss $L_{SWT}$. The evaluation was performed using the DIV2K test set in the ×4 super-resolution scenario. As shown in the results of Table 2, when the GCCA module was used, the PSNR, which focuses on objective quality, improved by 0.33, while the SSIM, which reflects subjective quality, increased by 0.021. This indicates that the introduction of the module contributes effectively to the restoration of image texture details in the reconstruction. With the gradual incorporation of StarRRDB, the GCCA module, the SFT layer, and wavelet loss $L_{SWT}$ into the model, both PSNR and SSIM values steadily improved. After introducing the GCCA module, the model can reallocate weights to semantically relevant parts, thereby focusing more on semantic texture details. The introduction of the SFT layer transfers semantically relevant conditional information to the super-resolution network, enhancing the realism of the generated textures. As shown in Figure 7, despite the blurry boundary between the buildings and grass, the model is still able to capture semantic information to some extent. Furthermore, after incorporating wavelet loss $L_{SWT}$, the model focuses more on high-frequency details, compensating for the traditional loss function’s inadequacy in capturing these details. Overall, the introduction of these modules significantly enhances the model’s ability to capture details and improves the quality of the generated images, as validated by the experimental results in the table.

## 5. Conclusions

We propose a GAN-based single-image super-resolution method, SP-IGAN. By integrating the SFT layer and GCCA module into the dense residual network (RRDB) module, SP-IGAN effectively captures and extracts image features. Additionally, by incorporating extra semantic information, the model further aggregates contextual data, enhancing its ability to comprehend image structure. The GCCA module redistributes pixel weights, reducing computational redundancy, while the SFT layer strengthens the model’s dependence on the relationship between texture and category information. Moreover, the inclusion of wavelet loss $L_{SWT}$ in the total loss calculation enhances the recovery and reconstruction of high-frequency details in the image, further strengthening the network’s representation capability. Extensive experiments demonstrate that SP-IGAN outperforms state-of-the-art methods across multiple public datasets, achieving superior PSNR and SSIM scores. It significantly improves texture consistency in region-specific categories and enhances subjective visual quality in single-image super-resolution reconstruction.

Future work can further explore single-image super-resolution methods that incorporate prior information, such as utilizing multimodal priors by integrating text descriptions into the super-resolution model. Considering the varying demands for super-resolution images in different scenarios, we can also train the model using domain-specific datasets, which would significantly enhance the performance of single-image super-resolution in complex scenarios. This study optimizes the model by improving the generator network, and it also explores an alternative approach by working with the discriminator. By introducing a semantic information extraction module, the authenticity of super-resolution images can be better assessed, and distributed learning can help improve the understanding of semantic structures. This approach not only reduces the computational cost of the generative model but also makes the model more lightweight and easier to integrate. This direction is worth further investigation.

## Figures and Tables

**Table 1:** A quantitative comparison is conducted between the SP-IGAN model and recent SOTA super-resolution methods, most of which are based on GANs. The model with the best performance is highlighted in red, while the second-best is marked in blue.

**Model**		DF2K		OST300		Set5		BSD100	
		**PSNR**	**SSIM**	**PSNR**	**SSIM**	**PSNR**	**SSIM**	**PSNR**	**SSIM**
ESRGAN [14]		25.45	0.7570	24.87	0.7221	26.10	0.7781	23.22	0.7064
SFTGAN [29]		25.53	0.7981	25.80	0.7881	26.82	0.7970	24.53	0.7153
Real-ESRGAN [19]		26.88	0.8569	26.63	0.8553	27.55	0.8225	24.40	0.7521
BSRGAN [17]	×2	26.21	0.8591	26.01	0.8621	26.12	0.8379	24.39	0.7560
FxSR-PD [31]		27.50	0.8811	27.20	0.8755	27.64	0.8530	25.36	0.7890
IG-CFAT [40]		26.02	0.8520	25.45	0.7813	26.91	0.8024	24.11	0.7559
ours		27.22	0.8951	27.31	0.8600	27.74	0.8561	26.01	0.7611
ESRGAN [14]		22.56	0.6553	22.22	0.6844	23.65	0.6849	20.89	0.6281
SFTGAN [29]		22.98	0.7255	22.82	0.7125	24.71	0.7051	21.74	0.6520
Real-ESRGAN [19]		24.65	0.8200	25.10	0.8251	25.47	0.8091	23.51	0.7222
BSRGAN [17]	×4	24.05	0.8012	24.35	0.8412	24.99	0.7538	22.97	0.6588
FxSR-PD [31]		25.15	0.8320	25.22	0.8505	25.66	0.8295	23.69	0.7583
IG-CFAT [40]		24.19	0.7998	23.95	0.7764	24.22	0.7873	22.59	0.7023
ours		25.20	0.8563	25.18	0.8520	25.51	0.8460	23.90	0.7619
ESRGAN [14]		22.16	0.6463	21.35	0.5979	21.85	0.6251	20.66	0.5830
SFTGAN [29]		22.75	0.7007	23.35	0.6896	24.01	0.6868	21.12	0.6258
Real-ESRGAN [19]		24.56	0.7890	23.99	0.8036	25.03	0.7735	23.05	0.7272
BSRGAN [17]	×8	23.85	0.7781	24.03	0.7890	24.47	0.7538	22.97	0.6588
FxSR-PD [31]		24.95	0.8097	25.12	0.8121	25.06	0.8055	23.28	0.7351
IG-CFAT [40]		23.91	0.7769	23.85	0.7534	24.10	0.7624	22.31	0.6821
ours		24.82	0.8211	24.91	0.8092	25.35	0.8350	23.58	0.7400

**Table 2:** Ablation experiments of SP-IGAN in the ×4 super-resolution model. The model with the best performance is highlighted in red, while the second-best is marked in blue.

	StarRRDB	GCCA	SFT	$L_{\mathrm{SWT}}$	PSNR ↑	SSIM ↑
1	*×*	*×*	*×*	*×*	24.65	0.8200
2	✓	*×*	*×*	*×*	24.68	0.8211
3	✓	✓	*×*	*×*	25.01	0.8421
4	✓	✓	✓	*×*	25.10	0.8489
5	✓	✓	✓	✓	25.20	0.8563

## Data Availability

The original contributions presented in this study are included in the article. Further inquiries can be directed to the corresponding author.
